# Effects of Heterologous Expression of Genes Related L–Malic acid Metabolism in *Saccharomyces uvarum* on Flavor Substances Production in Wine

**DOI:** 10.3390/foods13132038

**Published:** 2024-06-27

**Authors:** Ping Li, Wenjun Song, Yumeng Wang, Xin Li, Shankai Wu, Bingjuan Li, Cuiying Zhang

**Affiliations:** 1 College of Biotechnology and Food Science, Tianjin University of Commerce, Tianjin 300134, China; 2 Key Laboratory of Industrial Fermentation Microbiology, Ministry of Education, Tianjin Key Laboratory of Industrial Microbiology, College of Biotechnology, Tianjin University of Science and Technology, Tianjin 300457, China

**Keywords:** wine, heterologous expression, L–malic acid, higher alcohols, esters

## Abstract

During malolactic fermentation (MLF) of vinification, the harsh L–malic acid undergoes transformation into the milder L–lactic acid, and via decarboxylation reactions it is catalyzed by malolactic enzymes in LAB. The use of bacterial malolactic starter cultures, which usually present challenges in the industry as the suboptimal conditions after alcoholic fermentation (AF), including nutrient limitations, low temperatures, acidic pH levels, elevated alcohol, and sulfur dioxide concentrations after AF, lead to “stuck” or “sluggish” MLF and spoilage of wines. *Saccharomyces uvarum* has interesting oenological properties and provides a stronger aromatic intensity than *Saccharomyces cerevisiae* in AF. In the study, the biological pathways of deacidification were constructed in *S. uvarum*, which made the *S. uvarum* carry out the AF and MLF simultaneously, as different genes encoding malolactic enzyme (*mleS* or *mleA*), malic enzyme (*MAE2*), and malate permease (*melP* or *MAE1*) from *Schizosaccharomyces pombe*, *Lactococcus lactis*, *Oenococcus oeni*, and *Lactobacillus plantarum* were heterologously expressed. For further inquiry, the effect of L–malic acid metabolism on the flavor balance in wine, the related flavor substances, higher alcohols, and esters production, were detected. Of all the recombinants, the strains WYm1S_N_ with coexpression of malate permease gene *MAE1* from *S. pombe* and malolactic enzyme gene *mleS* from *L. lactis* and WYm1m2 with coexpression of gene *MAE1* and malate permease gene *MAE2* from *S. pombe* could reduce the L–malic acid contents to about 1 g/L, and in which the mutant WYm1S_N_ exhibited the best effect on the flavor quality improvement.

## 1. Introduction

The winemaking process usually involves alcoholic fermentation (AF) performed by *Saccharomyces cerevisiae* or other non-*Saccharomyces* yeasts and malolactic fermentation (MLF) driven by lactic acid bacteria (LAB) for most red and some white wines [1,2,3]. Grapes and certain wines undergoing AF harbor a diverse array of organic acids that exert a profound influence on the overall quality and flavor profile of the wine. Among these organic acids, malic acid and tartaric acid collectively constitute a substantial majority, ranging from 70% to 90%. Tartaric acid is regarded as the main contributor to wine acidity and was not metabolized by grape berry cells via respiration in the same manner as malic acid, predominantly existing in the wine matrix as potassium tartrate and potassium hydrogen tartrate, with only a minor fraction susceptible to conversion into lactic and acetic acid during MLF. Specifically, the harsh and astringent malic acid exhibits instability and is susceptible to rapid decomposition by microorganisms, ultimately leading to quality-related concerns [4,5,6,7,8]. It is imperative to conduct the pivotal process of MLF, during which the tart L–malic acid undergoes transformation into the milder L–lactic acid and carbon dioxide via a decarboxylation reaction catalyzed by malolactic enzymes in LAB, enhancing organoleptic balance and bolstering bacteriological stability in the wine. Hence, the malic acid content serves as a crucial indicator of fermentation completion, with a widely accepted guideline specifying that wines should have a malic acid content of less than 1 g/L [9,10]. The MLF process, on the other hand, presents challenges in terms of control due to various suboptimal physicochemical conditions, including nutrient limitations, low temperatures, acidic pH levels, elevated alcohol, and sulfur dioxide concentrations, competition with yeast and other bacterial strains, as well as susceptibility to bacteriophage infections. Additionally, MLF can give rise to undesirable metabolites, such as biogenic amines and ethyl carbamate by LAB [11,12,13].

Several approaches including blending, carbonate additions, precipitation, dilution, and carbonic maceration have been carried out to reduce the acidity of grape must or wine [14,15,16]. *Saccharomyces* are unable to efficiently metabolize malate due to their deficiency in an active malate permease responsible for the transport of extracellular malate [17]. During MLF, LAB facilitate deacidification by catalyzing the conversion of L-malate into L-lactate and CO_2_, which is chiefly mediated through malolactic enzyme (MLE) encoding by the gene *mleS* or *mleA*, and the malate transporter encoded by the gene *mleP* [18,19]. *Schizosaccharomyces pombe* has also demonstrated significant capability in reducing malic acid levels. *S. pombe* converted L-malate from must into ethanol and CO_2_, which is facilitated by the active transport of L–malic acid into the cells using an H^+^-symport system, driven by the activity of the malate permease (MAE1). Subsequently, the cytosolic malic enzyme (MAE2) catalyzes the oxidative decarboxylation of L-malate into pyruvate and CO_2_; pyruvate is further metabolized into ethanol and other flavors [20,21]. Therefore, the researchers endeavored to engineer malolactic pathways in *S. cerevisiae* by introducing constitutive expressions of the heterologous malolactic enzyme or malate transporter. Additionally, the recombinants exhibited varying degrees of malate decarboxylation during AF in wine. Bony et al. obtained a malolactic *S. cerevisiae* through the coexpression of the *Sz. pombe* malate transport gene (*MAE1*) and the *Lactococcus lactis* malolactic enzyme gene (*mleS*) under the control of promoter *PGK1*, leading to efficient decarboxylation 3 g/L of malate within a 3-day period [22]. Volschenk et al. demonstrated that a strain of *S. cerevisiae* containing the *Sz. pombe* malate permease (*MAE1*) and malic enzyme (*MAE2*) genes integrated in the genome efficiently degraded 5 g/L of L–malic acid in synthetic and Chenin Blanc grape musts [23]. Husnik et al. constructed a genetically stable industrial strain of *S. cerevisiae* by integrating a linear cassette containing the *Sz pombe* MAE1 and the *Oenococcus oeni* malolactic gene *mleA*, resulting in fully decarboxylated 5.5 g/L of malate in Chardonnay grape must during the AF [24]. *S. uvarum* has been shown to produce a stronger aromatic intensity than that produced by *S. cerevisiae*, although the regulatory mechanism underlying the heterologous genes in *S. uvarum* remains unclear. Moreover, it is noteworthy that the expression of the essential genes in *S. uvarum* metabolizes the malic acid, but at the same time it is bound to ruin the flavor balance of the wine [25,26,27].

Higher alcohols are one of the most important groups of aroma compounds that significantly influence the quality and flavor profiles of wine. Appropriate content and proportion of higher alcohols will lead to an alcoholic beverage that is mellow, soft, and plump, which are factors that coordinate the bouquet. An excessive level of higher alcohols will generate a strong fusel oil flavor, which may also potentially damage health [28,29]. It has been found that approximately 80% of higher alcohols in wine are generated during AF, concurrent with the reproduction and fermentation of wine yeasts. Higher alcohols are converted from α-ketoacids via decarboxylation by α-ketoacid decarboxylase and reduction of an aldehyde group by alcohol dehydrogenase. α-ketoacids involved can either be synthesized from pyruvate by the Harris pathway or from degradation of branched-chain amino acids (BCAAs) via the Ehrlich pathway [30,31]. Thus, it is important to acknowledge that alterations in malate or pyruvate levels resulting from the expression of essential genes related to malic acid degradation may also have an impact on the metabolism of higher alcohols and other related flavors.

In this study, the biosynthetic pathways for degradation of L–malic acid were constructed as the heterologous genes from *L. lactis*, *O. oeni*, *L. plantarum* (considered as the next-generation MLF fermented with the prospect of replacing *O. oeni*), and *S. pombe* were expressed or coexpressed in *S*. *uvarum.* Besides physiological characteristics and fermentation performances, including the growth curve and the content of ethanol, residual sugar, and organic acid, the higher alcohols and esters concentrations in wine were also detected to confirm the effect the heterologous gene expressions in *S. uvarum.*

## 2. Materials and Methods

### 2.1. Strains and Media

The strains (*Escherichia coli* DH5α, *S. uvarum*, *L. lactis*, *O. oeni*, *L. plantarum*, and *S. pombe*) used in this study are described in Table 1. The *E. coli* strain DH5α, which was used as a host for recombinant DNA manipulation, was grown at 37 °C in LB broth (1% NaCl, 1% tryptone, and 0.5% yeast extract); *L. lactis* was cultured via SGM17 medium (37.25 g/L M17 broth, 5 g/L sucrose, and 5.5 g/L glucose); *S. uvarum* and *S. pombe* were grown in YPD medium (20 g/L glucose, 10 g/L yeast extract, and 20 g/L bacto-peptone) at 30 °C; *L. plantarum* cells were propagated in MRS medium (2% glucose, 1.0% tryptone, 1.0% beef extract, 1.0% yeast extract, 0.2% K_2_HPO_4_, 0.1% Tween-80, 0.02% MgSO_4_·7H_2_O, 0.005% MnSO_4_·4H_2_O, 0.2% ammonium citrate, 0.5% sodium acetate, 50 mM NaHCO_3_) at 37 °C; MRS medium supplemented with 10% (*v/v*) tomato juice was used to enumerate *O. oeni* at 25 °C. All engineered strains are derivatives of *S. uvarum* WY1.

### 2.2. Construction of Plasmids

The plasmids (Yep352, pUC19-PGK, and pUG6) used in the current study are shown in Table 2. Plasmid pUC19-PGK and pUG6 were used as templates to obtain the promoter *PGK1* fragment and selection marker *KanMX* fragment, respectively. Yep352 was used as the backbone to construct the recombinant plasmids.

The recombinant plasmid Yep-P was constructed as previously reported. The fragments of genes *MAE1*, *MAE2*, *mleS_N_*, *mleA_J_*, *mleS_Z_* amplified from *S. uvarum*, *S. pombe*, *L. lactis*, *O. oeni*, and *L. plantarum* were cloned into Yep-P digested with *Xho*I to obtain the plasmid Yep-PE, Yep-Pm2, Yep-PS_N_, Yep-PA_J_, and Yep-PS_Z_, respectively. Similarly, the gene *MAE1* from *S. pombe* and genes *mleP* from *L. lactis*, *O. oeni*, and *L. plantarum* were inserted into Yep-P, constructing the recombinant plasmids Yep-Pm1, Yep-PP_N_, Yep-PP_J_, and Yep-PP_Z_, respectively. The fragments PGKp-MAE1-PGKt, PGKp-MAE2-PGKt, PGKp-mleS_N_-PGKt, PGKp-mleS_Z_-PGKt, and PGKp-mleA_J_-PGKt were cloned into Yep-PP_N_ at the *Sma*I restriction site, obtaining Yep-PP_N_E, Yep-PP_N_m2, Yep-PP_N_S_N_, Yep-PP_N_S_Z_, and Yep-PP_N_A_J_, respectively, and the fragments PGKp-MAE1-PGKt, PGKp-MAE2-PGKt, PGKp-mleS_N_-PGKt, PGKp-mleA_J_-PGKt, and PGKp-mleS_Z_-PGKt were cloned into Yep-Pm1 at the *Sma*I restriction site, resulting in Yep-Pm1E, Yep-Pm1m2, Yep-Pm1S_N_, Yep-Pm1A_J_, and Yep-Pm1S_Z._ Then, the selection marker *KanMX* was inserted into the recombinant plasmids after digestion of *Apa*I, the plasmids Yep-PEK, Yep-Pm2K, Yep-PS_N_K, Yep-PS_z_K, Yep-PA_J_K, Yep-Pm1K, Yep-PP_N_K, Yep-PP_Z_K, Yep-PP_J_K, Yep-PP_N_EK, Yep-PP_N_m2K, Yep-PP_N_S_N_K, Yep-PP_N_S_Z_K, Yep-PP_N_A_J_K, Yep-Pm1EK, Yep-Pm1m2K, Yep-Pm1S_N_K, Yep-Pm1A_J_K, and Yep-Pm1S_Z_K were constructed. 

### 2.3. Transformation Strategy

The transformation process was performed using the lithium acetate/PEG method. The experiment procedures, including cell culture, preparation, and transformation of competent cells, were carried out as previously reported [16]. The plasmids Yep-P, Yep-PEK, Yep-Pm2K, Yep-PS_N_K, Yep-PS_z_K, Yep-PA_J_K, Yep-Pm1K, Yep-PP_N_K, Yep-PP_Z_K, Yep-PP_J_K, Yep-PP_N_EK, Yep-PP_N_m2K, Yep-PP_N_S_N_K, Yep-PP_N_S_Z_K, Yep-PP_N_A_J_K, Yep-Pm1EK, Yep-Pm1m2K, Yep-Pm1S_N_K, Yep-Pm1A_J_K, and Yep-Pm1S_Z_K were transformed into *S. uvarum* WY1, and then the mutant strains WY0, WYE, WYm2, WYS_N_, WYS_Z_, WYA_J_, WYm1, WYP_N_, WYP_Z_, WYP_J_, WYP_N_E, WYP_N_m2, WYP_N_S_N_, WYP_N_S_Z_, WYP_N_A_J_, WYm1E, WYm1m2, WYm1S_N_, WYm1A_J_, and WYm1S_Z_.

### 2.4. Fermentation Conditions

The detailed procedures, encompassing grape acquisition, juice adjustment, preparation of yeast strains, and inoculation of *S*. *uvarum*, closely followed the protocols established by Li et al. [16]. The must was adjusted to 20.45 Brix and pH 3.4–3.6 by addition of sucrose and tartaric acid, respectively. A 250 mL autoclaved flask was filled with 190 mL of formulated must. The modified grape juice, following adjustments, underwent fermentation at 25 °C in a temperature-controlled environment. The presence of residual sugar at 0.5% indicated completion of the yeast fermentation process. All fermentations were performed in triplicate. 

### 2.5. Chemical Analysis

The growth curves of the parental strain and mutant strains were monitored using a Bioscreen Automated Growth Curves analysis system. The CO_2_ weight loss was measured at 12 h intervals using an analytical balance throughout the fermentation process. Following fermentation, the pH, ethanol concentration, and residual sugar content were determined using a pH meter, oenometer, and Brix hydrometer, respectively. L–malic acid, L–lactic acid, and other organic acids were identified using high-performance liquid chromatography (HPLC), while flavor compounds, including higher alcohols, ethyl acetate, and ethyl lactate, were analyzed via an Agilent 7890C GC (Santa Clara, CA, USA). The detailed experimental conditions were implemented as reported previously [32]. All analyses were conducted in triplicate. 

### 2.6. Real-Time Quantitative PCR (RT-qPCR)

The total RNA of *S. uvarum* was extracted using a Yeast RNAiso Kit (Takara Biotechnology, Dalian, China), followed by reverse transcription using a PrimeScript™ RT reagent Kit with gDNA Eraser (Perfect Real Time) (Takara Biotechnology, Dalian, China). The abundance of mRNAs encoding the target genes was determined by amplifying the genes using corresponding cDNAs as PCR templates. Changes in gene expression levels were evaluated by RT-qPCR employing an SYBR *Premix Ex Taq* II (Tli RNaseH Plus) (Takara Biotechnology, Dalian, China). The expression level of the target genes was normalized relative to the expression level of *UBC6* that served as a reference gene [33]. The results were quantitatively analyzed using the 2^−ΔΔCt^ method.

### 2.7. Statistical Analysis

Data were represented as the mean ± standard errors. Data analysis was conducted using Origin 9.0 and SPSS 24.0 statistical software. The differences between the mutant strains and parental strain were confirmed by Student’s *t*-test. Statistical significance was considered at *p* < 0.05.

## 3. Results and Discussion

**Figure 1:**
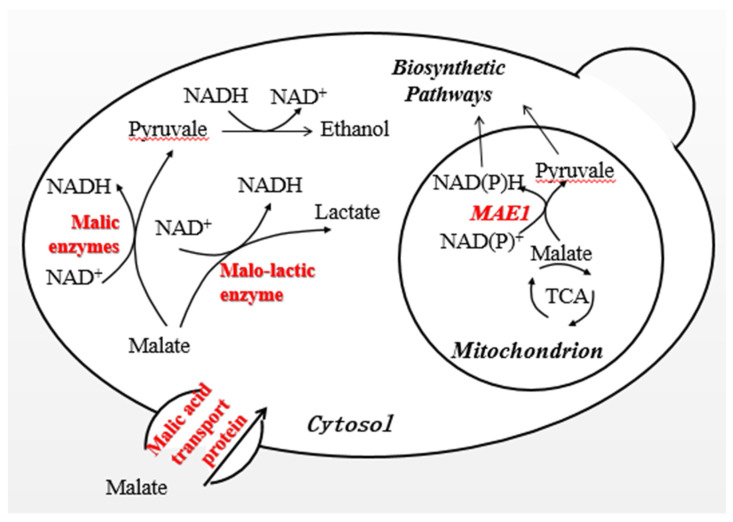
Biosynthetic pathway of L–malic acid metabolism in yeast.

During winemaking, LAB carry out the MLF to convert the harsh malic acid into supple and stable lactic acid and CO_2_ catalyzed by the malolactic enzyme (MLE), resulting in deacidification and improving the organoleptic balance of wine. When using bacterial malolactic starter cultures, LAB grow poorly and unpredictably in wine, which leads to “stuck” or “sluggish” MLF and spoilage of wines. Thus, performing both the AF and MLF with a single wine yeast strain is an absolute requirement for winemaking. In the current study, recombinant *S. uvarums* was transferred into malolactic activity genes to perform the MLF during AF, as shown in Figure 1. Following the method used for the introduction of a plasmid carrying a multicopy of regulatory or structural genes after codon optimization to test the gene regulation at molecular levels, a similar process was carried out to investigate the potential for *S. uvarum* [16]. 

### 3.1. Effect on L–Malic acid Degradation of Single-Gene Heterologous Expression in S. uvarum

**Figure 2:**
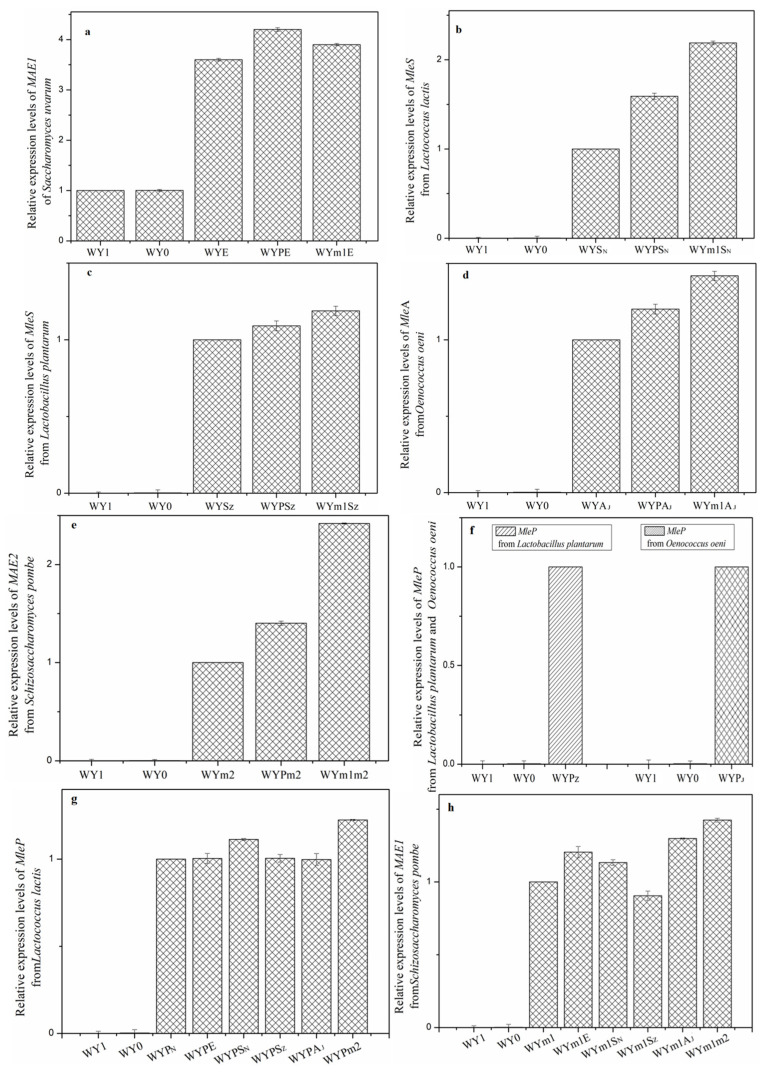
Determination of heterologous genes and *MAE1* expression levels in parental strain WY1 and recombinant strains. Data are the average of three independent experiments. Error bars represent ±SD. (**a**) Relative expression levels of *MAE1* gene in the mutants WYE, WYPE, WYm1E compared with the parental strain WY1. (**b**) Relative expression levels of *mleS* gene from *Lactococcus lactis* in the mutants WYPS_N_, WYm1S_N_, and parental WY1 compared with the mutant WYS_N_. (**c**) Relative expression levels of *mleS* gene from *Lactobacillus plantarum* in the mutants WYPS_Z_, WYm1S_Z_, and parental WY1 compared with the mutant WYS_Z_. (**d**) Relative expression levels of *mleA* gene from *Oenococcus oeni* in the mutants WYPA_J_, WYm1A_J_, and parental WY1 compared with the mutant WYA_J_. (**e**) Relative expression levels of *MAE2* gene from *Schizosaccharomyces pombe* in the mutants WYPm2, WYm1m2, and parental WY1 compared with the mutant WYm2. (**f**) Relative expression levels of *mleP* gene from *Lactobacillus plantarum* in the parental WY1 compared with the mutant WYP_Z_, and relative expression levels of *mleP* gene from *Oenococcus oeni* in the parental WY1 compared with the mutant WYP_J_. (**g**) Relative expression levels of *mleP* gene from *Lactococcus lactis* in the mutants WYPE, WYPS_N_, WYPS_Z_, WYPA_J_, WYPm2, and parental WY1 compared with the mutant WYP_N_. (**h**) Relative expression levels of *MAE1* gene from *Schizosaccharomyces pombe* in the mutants WYm1E, WYm1S_N_, WYm1S_Z_, WYm1A_J_, WYm1m2, and parental WY1 compared with the mutant WYm1.

**Figure 3:**
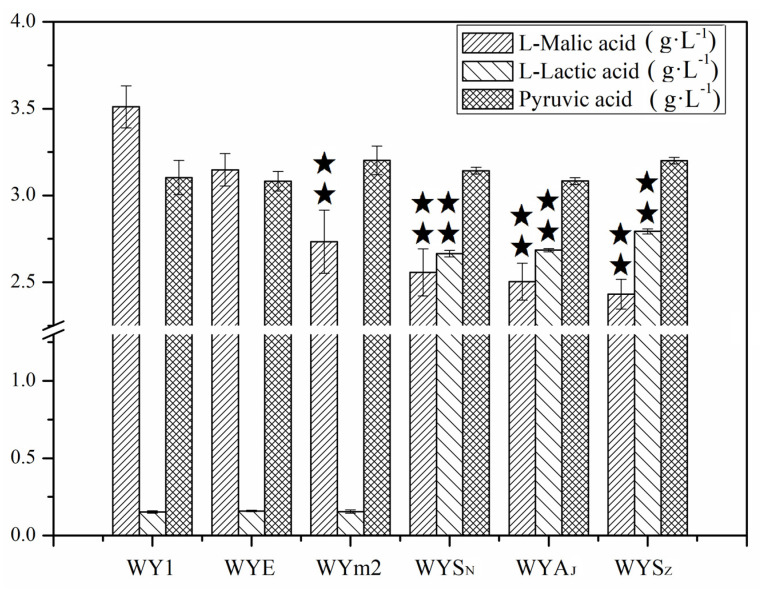
Concentrations of L–malic acid, L–lactic acid, and pyruvic acid in the mutants WYE, WYm2, WYS_N_, WYS_Z_, WYA_J_, and the parental WY1. Error bars represent the SD of the average values. Statistical significance is denoted as ★★ = *p* < 0.01.

The malic enzyme encoded by gene *MAE1* in *S. uvarum* and gene *MAE2* in *S. pombe* catalyzes the oxidative decarboxylation of malate to pyruvate and CO_2_. The malolactic enzyme encoded by gene *mleS* in *L. lactis* and *L. plantarum*, and gene *mleA* in *O. oeni* are devoted to transform the L–malic acid to L–lactic acid and CO_2_ [21,34,35]. Thus, to explore the potential gene regulation on L–malic acid degradation, the genes were expressed in *S. uvarum*, respectively, under the controls of the promoter *PGK1*, constructing the mutant strains WYE, WYm2, WYS_N_, WYS_Z_, and WYA_J_. To verify the expression of the heterologous genes in the mutants, we quantified their relative expression levels using RT-qPCR. The results are depicted in Figure 2. The relative expression levels of genes *mleS*, *mleA,* or *MAE2* in the mutants exhibited significant improvements compared to those in the parental strain, respectively (*p* < 0.05). Consequently, the heterologous genes were cloned and expressed accurately in *S. uvarum* WY1. After AF, the concentrations of L–malic acid, L–lactic acid, and pyruvic acid were detected, the result is shown in Figure 3. The L–malic acid content by the mutant strains WYE, WYm2, WYS_N_, WYA_J_, and WYS_z_ were 3.148, 2.733, 2.557, 2.504, and 2.432 g/L, thus reduced by 10.36%, 22.16%, 27.18%, 28.68%, and 30.73% compared with that of the parental strain (3.511 g/L, *p* < 0.05). And the L–lactic acid production of WYE and WYm2 showed no significant changes compared to the wild-type strain, but that of WYS_N_, WYA_J_, and WYS_z_ was 2.665, 2.684, and 2.793 g/L, which were 16~18-fold higher than that produced by the parental strain (0.152 g/L, *p* < 0.05). In addition, the pyruvic acid production of the engineered strains showed no significant difference compared with that of the parental strain. This discrepancy constitutes a primary factor contributing to the variation in fermentation yield of higher alcohols between the mutant strains and the parental counterpart. 

**Figure 4:**
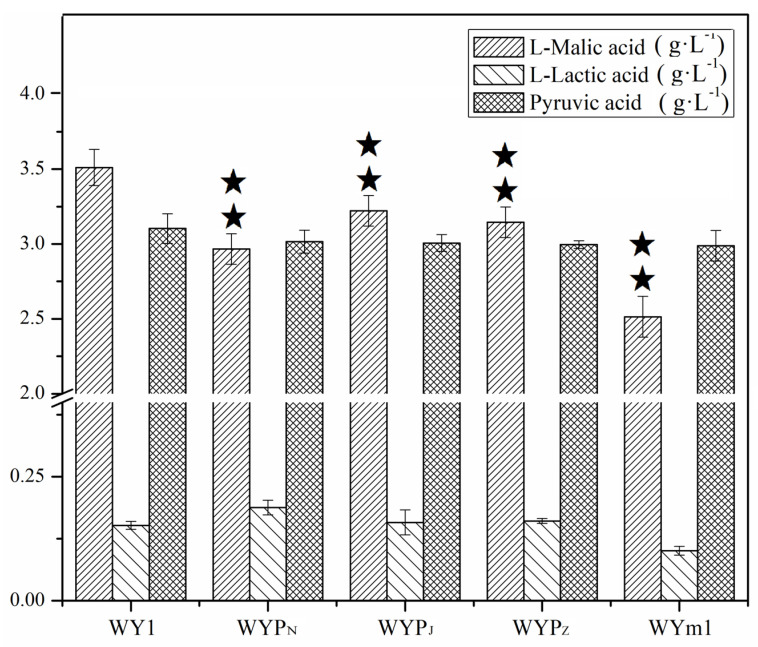
Concentrations of L–malic acid, L–lactic acid, and pyruvic acid in the mutants WYP_N_, WYP_Z_, WYP_J_, WYm1, and the parental WY1. Error bars represent the SD of the average values. Statistical significance is denoted as ★★ = *p* < 0.01.

**Figure 5:**
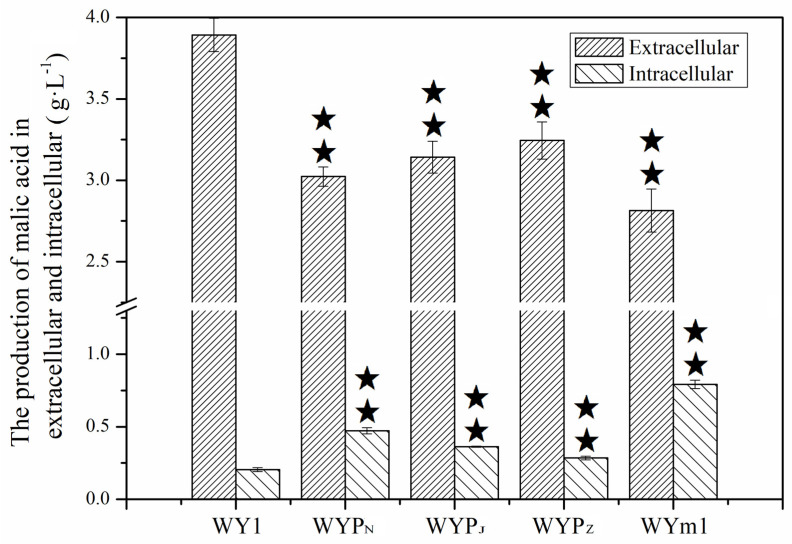
The extracellular and intracellular production of L–malic acid in the mutants WYP_N_, WYP_Z_, WYP_J_, WYm1, and the parental WY1. Error bars represent the SD of the average values. Statistical significance is denoted as ★★ = *p* < 0.01.

*Saccharomyces* exhibit limited efficiency in malate metabolism, attributed to their lacking an active malate permease responsible for extracellular malate transport [17,36,37]. Malate permease mleP in LAB and MAE1 in *S. pombe* are the active malate transporters from extracellular to intracellular compartments [21,38]. Consequently, the *mleP* genes from *L. lactis*, *L. plantarum*, and *O. oeni*, along with the *mae1* gene from *S. pombe*, were cloned and expressed in *S. uvarum* under the regulation of the *PGK1* promoter, respectively. The mutants WYP_N_, WYP_Z_, WYP_J_, and WYm1 were obtained. The significant improvements observed in the relative expression levels of *mleP* or *MAE1*, as shown in Figure 1, in the mutants indicate the successful expression of the exogenous genes in *S. uvarum*. As shown in Figure 4, The L–malic acid contents of the wine fermented by WYP_N_, WYP_Z_, WYP_J_, and WYm1 were 2.967, 3.145, 3.222, and 2.514 g/L, respectively, indicating a decrease of 15.51%, 10.42%, 8.23%, and 28.41% compared to that produced by the parental strain (*p* < 0.05). There were no significant changes in the contents of lactic acid and pyruvate produced by both the mutants and the parental strain. Additionally, to assess the mutants’ capacity for malate transport between extracellular and intracellular compartments, the fermentation experiment was conducted by inoculating simulated grape juice. The production of intracellular L–malic acid was measured as depicted in Figure 5. The intracellular L–malic acid contents in the mutants WYP_N_, WYP_Z_, WYP_J_, and WYm1 were 0.473, 0.362, 0.285, and 0.792 g/L, respectively, indicating a significant improvement compared to the parental strain. This demonstrates that the heterologous expression of genes encoding malate permease significantly influences the transport of malic acid from extracellular to intracellular compartments in *S. uvarum*.

In LAB, the malolactic enzyme converts L-malate to L-lactate without free intermediates or net reduction of NAD^+^; thus, the heterologous expressions of genes encoding malolactic enzyme in WYS_N_, WYS_z_, and WYA_J_ lead to the degradation of L–malic acid, and the L-lactate contents were increased significantly. The malic enzymes *MAE1* in *S. uvarum* and *MAE2* in *S. pombe* are responsible for catalyzing the oxidative decarboxylation of L-malate to pyruvate and CO_2_. Although the L-malate production was slightly reduced, pyruvate production of WYE and WYm2 were relatively similar to that of the parental strain WY1, which was probably due to further metabolization of pyruvate to ethanol/lactic acid and other flavor compounds under fermentative conditions. The expression of malolactic enzyme from LAB and malic enzyme from *S. uvarum* and *S. pombe* in *S. uvarum* could not effectively degrade malic acid to standard content (≤1 g/L), which was in accordance with the results of the genes expressed in *S. cerevisiae*. These failed attempts contributed to the absence of a malate transporter in *S. uvarum* as in *S. cerevisiae* [35].

Despite reports indicating that the degradation of L–malic acid primarily depends on copies of the malate permease gene in LAB [19,34,35], the expression of the gene *mleP* in *L. lactis*, *L. plantarum*, and *O. oeni*, or the gene *MAE1* in *S. pombe*, which encodes for malic enzyme in *S. uvarum* did not result in further malate decarboxylation compared to the heterologous expression of malolactic enzyme in LABs or malic enzyme in *S. pombe*. Perhaps the lack of enhancement in malate decarboxylation in *S. uvarum* may be attributed to the metabolic regulation of its cells. Conversely, while malic acid can be transported into the cell via malate permease, the cell exhibits limited capacity and lacks efficient enzymes for malic acid degradation. Hence, to facilitate the efficient metabolism of malic acid in *S. uvarum*, a synergistic action of both malate permease and malate-degrading enzymes (such as malolactic enzyme or malic enzyme) is indispensable.

### 3.2. Effects of Coexpression of Malate Permease mleP from L. lactis with Different Malolactic Enzyme or Malic Enzyme Genes in S. uvarum on the Degradation of L–Malic Acid

**Figure 6:**
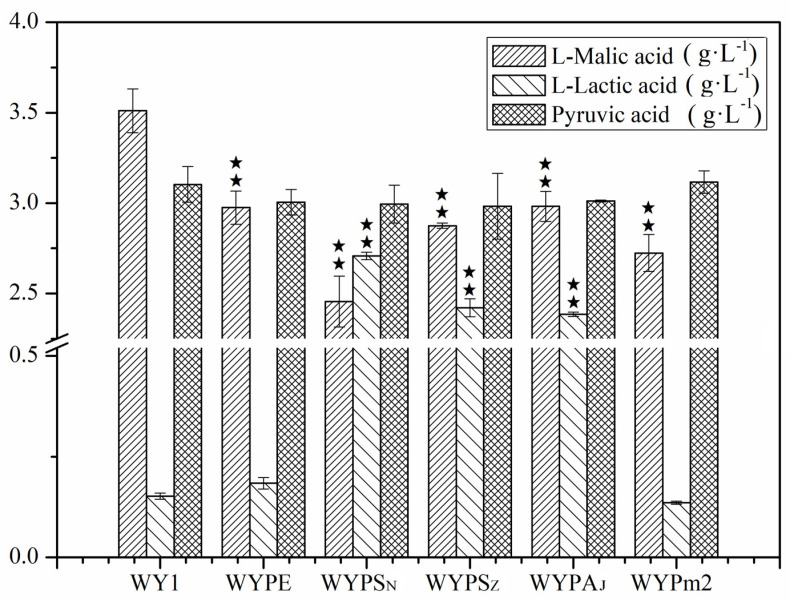
Concentrations of L–malic acid, L–lactic acid, and pyruvic acid in the mutants WYPE, WYPm2, WYPS_N_, WYPS_Z_, WYPA_J_, and the parental WY1. Error bars represent the SD of the average values. Statistical significance is denoted as ★★ = *p* < 0.01.

The heterologous expression of single-gene encoding malate permease, malolactic enzyme, or malic enzyme in *S. uvarum* proves ineffective in metabolizing malic acid, even upon overexpression of the native gene *MAE1*. Consequently, the malate transport gene *mleP* from *L. lactis* was heterologously expressed in *S. uvarum* in conjunction with the malolactic enzyme gene (*mleS* in *L. lactis* and *L. plantarum*, *mleA* in *O. oeni*) or malic enzyme gene *MAE1* in *S. uvarum* and *MAE2* in *S. pombe*, respectively. The mutants WYPS_N_, WYPS_Z_, WYPA_J_, WYPE, and WYPm2 were constructed. After fermentation, the results, as shown in Figure 6, revealed that the production of L–malic acid by the mutant strains WYPS_N_, WYPS_Z_, WYPA_J_, WYPE, and WYPm2, was 2.455, 2.875, 2.982, 2.975, and 2.724 g/L, respectively, indicating a reduction of 30.09%, 18.11%, 15.066%, 15.28%, and 22.43%, respectively, compared to that produced by the parental strain (*p* < 0.05). Additionally, the concentration of L–lactic acid in the mutant strains WYPS_N_, WYPS_Z_, WYPA_J_, was 2.708, 2.421, and 2.385 g/L, respectively, showing an increase of approximately 15–17 fold compared to that in the parental strain (*p* < 0.05). Conversely, there were no significant changes observed in the mutant strains WYPE, WYPm2, and the parental strain WY1. The results demonstrated the combination of gene mleP of *L. lactis* with different malolactic enzyme or malic enzyme genes in *S. uvarum* did not lead to further degradation of L–malic acid compared with the single gene expressed in *S. uvarum.* The inefficiency observed may stem from the fact that the decarboxylation system of L–malic acid in *L. lactis* necessitates the collaborative action of malate permease, malolactic enzyme, and other regulatory proteins. It was reported that the activator protein MleR serves as a positive regulator crucial for the expression of malolactic enzyme and the initiation of MLF in LABs [37]. Consequently, the coexpression of MleP with malolactic enzyme or malic enzyme genes in *S. uvarum* have failed to adequately complete the MLF process, potentially also due to the differential expression of the heterologous genes in *S. uvarum*. Further research is warranted to delve deeper into these observations.

### 3.3. Effects of Coexpression of Malate Permease MAE1 from S. pombe with Different Malolactic Enzyme or Malic Enzyme Genes in S. uvarum on the Degradation of L–Malic Acid

**Figure 7:**
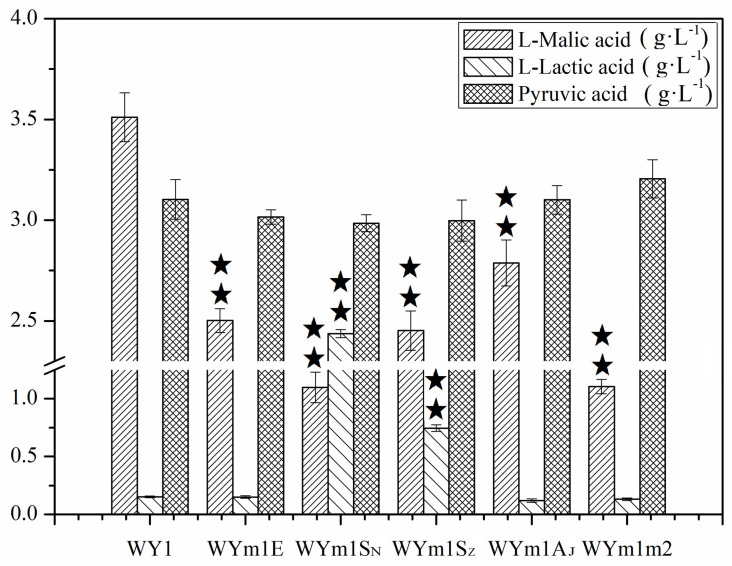
Concentrations of L–malic acid, L–lactic acid, and pyruvic acid in the mutants WYPE, WYm1m2, WYm1S_N_, WYm1S_Z_, WYm1A_J_, and the parental WY1. Error bars represent the SD of the average values. Statistical significance is denoted as ★★ = *p* < 0.01.

*S. pombe* demonstrates the capability to fully convert L-malate into ethanol and CO_2_ during fermentation in wine, thereby contributing to deacidification. The core system involved in this process consists of the malate transport (encoded by gene *MAE1*) and the malic enzyme (encoded by gene *MAE2*). In *S. uvarum*, the expression of the gene *MAE1* was combined with malolactic enzyme genes (*mleS* from *L. lactis*, *L. plantarum*, and *mleA* in *O. oeni*), or malic enzyme genes *MAE1* in *S. uvarum* and *MAE2* in S. *pombe*, resulting in the generation of mutants WYm1S_N_, WYm1A_J_, WYm1S_Z_, WYm1E, and WYm1m2. As depicted in Figure 7, the L-malate concentrations of WYm1E, WYm1m2, WYm1S_N_, WYm1A_J_, and WYm1S_Z_ were 2.502, 1.105, 1.098, 2.788, and 2.452 g/L, respectively, representing reductions of 28.75%, 68.52%, 68.74%, 20.61%, and 30.16%, respectively, compared to those produced by the parental strain WY1 (*p* < 0.05). Moreover, the L–lactic acid concentration increased in mutant strains WYm1S_N_ and WYm1S_Z_ to 2.437 and 0.745 g/L, respectively, while it decreased by 20.53% in strain WYm1A_J_, respectively. These findings demonstrate that the heterologous expression of these genes had a beneficial effect on malate degradation, with strains WYm1m2 and WYm1S_N_ showing the best results, nearly reaching the defined threshold of malic acid (<1 g/L).

The coexpression of malate transport MAE1 from *S. pombe* with different malolactic enzymes or malic enzymes genes in *S. uvarum* exhibited different effects on the degradation of L–malic acid in wine. Although *O. oeni* carried the much greater ability for deacidification than any other LAB during MLF in wine, the gene *mleA* showed little effect on metabolizing L–malic acid with the malate transport *MAE1* from *S. pombe* in *S. uvarum*. The result was inconsistent with the research reported by Husnik et al., in which the industrial strain of *S. cerevisiae* coexpressing the *S. pombe* malate permease gene (*MAE1*) and the *O. oeni* malolactic gene (*mleA*) could fully decarboxylated 5.5 g/L of malate in Chardonnay grape must and produced equimolar amounts of lactate [24]. Besides the alternative expression of the heterologous gene in *S. uvarum*, it also is probably due to the NAD-dependent malolactic enzyme that is subject to catabolite repression in *S. uvarum* exhibiting low substrate affinity. Furthermore, the decreased L–lactic acid concentration in strain WYm1A_J_ may be attributed to more intracellular malate transported from the extracellular environment by malate permease, which was metabolized by the native malic enzyme rather than the malolactic enzyme. In addition, the result, which showed that the combination of *S. pombe* malate permease gene (*MAE1*) and malic enzyme gene (*MAE2*) or *L. lactis* malolactic gene (*mleS*) could effectively degrade malate production to about 1 g/L during AF, was in accord with Volschenk and colleagues’ research, in which the mutant *S. cerevisiae* expressing the *S. pombe MAE1* and *MAE2* genes degraded 8 g/L malate in a glycerol–ethanol medium within 7 days, and the recombinant malolactic *S. cerevisiae* (*S. pombe MAE1* and *L. lactis mleS* genes) could ferment 4.5 g/L of malate in a synthetic grape must within 4 days [23]. *L. plantarum* strains were capable of surviving harsh wine conditions and displayed a more diverse enzyme profile than *O. oeni*, whereas coexpression of *S. pombe MAE1* and *L. plantarum mleS* genes did not completely metabolize the malate content to about 1 g/L. The results also demonstrated that the main cause that the LAB completed the MLF in wine is that the genes resisted the harsh wine conditions. The mechanisms underlying these effects, however, require further research.

### 3.4. Effects of the Gene Expressions in S. uvarum on Higher Alcohols and Other Flavor Substances Production

Currently, research on the regulation of genes involved in deacidification in *S. cerevisiae* during alcoholic fermentation (AF) largely concentrates on studying individual substances such as malic acid, precursors, or metabolites within a single metabolic pathway, neglecting the balance of wine flavor. Higher alcohols and esters, crucial factors determining the sensory characteristics and flavor profile quality in wine, were detected to assess the impact of heterologous gene expressions in S. uvarum, as outlined in Table 3. The production of flavor substances was not affected by the overexpression of the *MAE1* gene encoding malic enzyme in *S. uvarum*. In mutant WYm2, with heterologous expression of the gene *MAE2* from *S. pombe*, isoamyl alcohol and ethyl acetate levels were 212.105 and 28.520 mg/L, reflecting increases of 5.25% and 22.40%, respectively, compared to the parental strain WY1 (isoamyl alcohol content: 201.530 mg/L; ethyl acetate production: 23.301 mg/L, *p* < 0.05). In addition, the production of isobutyl alcohol, isoamyl alcohol, ethyl acetate, and ethyl lactate were variably affected as the heterologous expression of malolactic enzyme genes. For example, the isobutyl alcohol contents of strains WYS_N_, WYA_J_, WYS_Z_ were 29.955, 31.832, and 32.375 mg/L, showing reductions of 15.27%, 9.96%, and 8.43%, respectively, compared to the parental strain WY1 (35.354 mg/L). The isoamyl alcohol contents of the mutants were 176.282, 189.205, and 191.472 mg/L, representing decreases of 12.53%, 6.12%, and 5.04%, respectively, compared to the parental strain WY1 (201.53 mg/L). The ethyl acetate production by the mutants were 21.716, 21.573, and 21.630 mg/L, exhibiting reductions of 6.80%, 7.42%, and 7.17%, respectively, compared to the parental strain WY1 (23.301 mg/L). The ethyl lactate levels in the mutants increased to 11.115, 5.065, and 6.130 mg/L, respectively. Additionally, the isobutyl alcohol, isoamyl alcohol, and ethyl acetate contents produced by WYP_N_ (with heterologous expression of gene *mleP* encoding malate permease from *L. lactis*) were 36.335, 217.858, and 27.482 mg/L, increased by 2.78%, 8.10%, and 17.90%, respectively. Lastly, those produced by mutant WYm1 (heterologous expression of gene *MAE1* encoding malate permease from *S. pombe*), were 45.244, 223.662, and 25.915 mg/L, reflecting improvements of 27.98%, 10.98%, and 11.22%, respectively, compared to those produced by the parental strain WY1.

**Figure 8:**
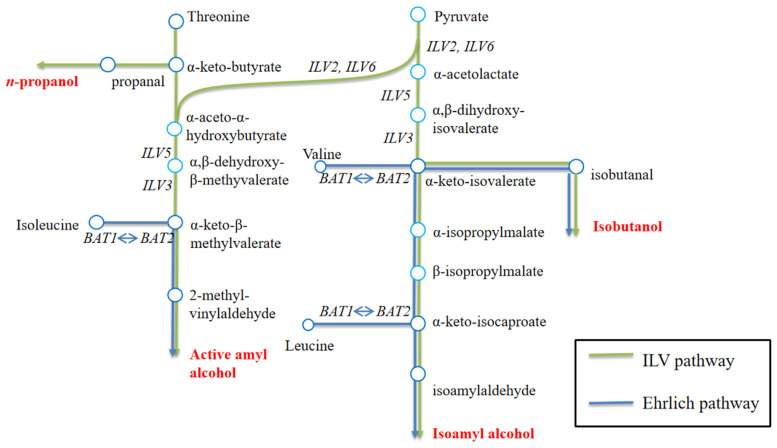
Metabolic pathways of higher alcohols in yeast.

The heterologous expression of the malic enzyme gene *MAE2* from *S. pombe* in *S. uvarum* WY1 resulted in a significant increase in the content of ethyl acetate and isoamyl alcohol in wine. This may be attributed to the fact that expression of the malic enzyme gene *MAE2* in *S. uvarum* WY1 resulted in the conversion of malic acid to pyruvic acid. Subsequently, a portion of the pyruvic acid was metabolized to produce acetaldehyde, which further formed acetic acid and was then esterified with ethanol to generate ethyl acetate. Another portion of pyruvic acid was channeled through a series of enzymes in the ILV pathway, leading to the formation of α-keto acid, eventually resulting in the production of isoamyl alcohol (as shown in Figure 8). The malolactic enzyme encoded by gene *mleS* or *mleA* degrades malic acid to lactic acid. And the lactic acid reacts with ethanol to produce ethyl lactate, effectively slowing the metabolic flow from malic acid to pyruvic acid in *S. uvarum*. Thus, the production of isobutyl alcohol and isoamyl alcohol were reduced after pyruvic acid metabolism. Additionally, alterations in L–malic acid levels could affect the tricarboxylic acid cycle, and then impacts the metabolism of compounds such as pyruvic acid and α-ketoglutaric acid in *S. uvarum*, thereby leading to variations in higher alcohols and ethyl acetate concentration in wine. Malate permease plays a crucial role in supporting MLF. We suspect that that the heterologous expression of gene *mleP* promoted the L–malic acid transfer from extracellular to intracellular, induced the expression of malic enzyme in *S. uvarum* and facilitated the conversion of malate to pyruvate. This process, in turn, leads to an increase in levels of higher alcohols. Additionally, malate permease-regulated malate transporter proteins exhibit permeability to other extracellular dibasic acids, such as α-ketoglutarate. This dual function affects the tricarboxylic acid cycle in *S. uvarum*. In yeast cells, aminotransfer between amino acids and their corresponding α-keto acids is catalyzed by branched-chain amino acid aminotransferases (BCAATases) in both the mitochondria and cytoplasm. This process synthesizes leucine, isoleucine, and valine using glutamate and α-ketoglutarate as donor and acceptor, respectively. Furthermore, the amino acids and α-ketoglutarate are utilized to produce the corresponding α-keto acids by transaminases. These α-keto acids, such as α-ketoisovaleric acid, hexanoic acid, and α-ketoisocaproic acid, undergo decarboxylation and dehydrogenation to produce isobutanol and isopentyl alcohol [38,39,40].

Therefore, the coexpression of the malate permease gene and either the malolactic enzyme gene or the malic enzyme gene in the mutants exhibited varying effects on the content of higher alcohols and esters in wine. The malate transporter facilitated the transfer of L-malate and α-ketoglutarate from the extracellular environment to the intracellular space. The transportation promotes the expression of malic enzyme and malolactamase genes (as shown in Figure 2), leading to the degradation of malic acid into lactic acid or pyruvic acid. Consequently, the metabolism of higher alcohols and esters were affected differently. Of the mutants, strain WYm1S_N_ exhibited the best effect whether on higher alcohols levels or on esters production. The increased lactic acid content led to an ethyl lactate concentration rising from approximately 0 to about 13.990 mg/L. This finding is essentially consistent with our previous report on wine fermented by yeast and LAB simultaneously [16]. Additionally, the production of higher alcohols was effectively reduced. Excessive levels of higher alcohols can contribute to the generation of a strong fusel oil flavor and pose potential health problems.

### 3.5. Effects of the Gene Expressions in S. uvarum on Growth and Fermentation Performance

**Figure 9:**
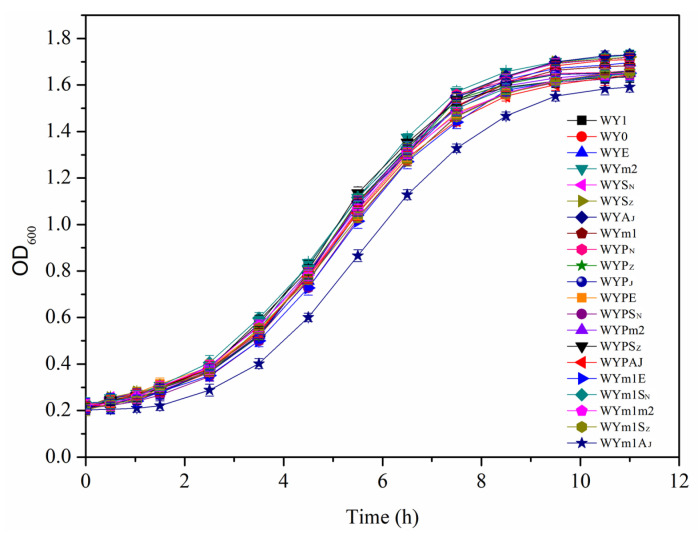
Growth curve of the mutants and the parental strain WY1.

**Figure 10:**
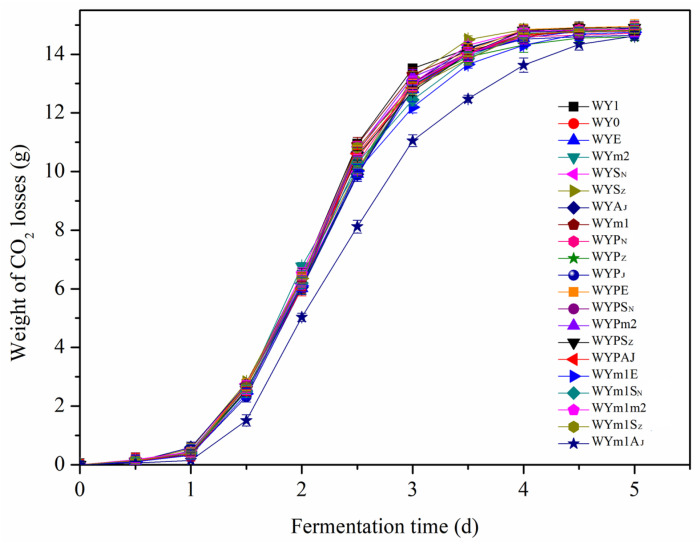
The cumulative CO_2_ weightlessness of the wine fermented by the parental strain and the mutants.

A stable fermentation performance of the mutant strain was closely related with liquor yield, fermentation period, and flavor quality of the wine in industrial fermentation. Thus, the physiological characteristic and fermentation performances, such as ethanol, residual sugar, and organic acid of wine, were determined to investigate the effects of heterologous expressions of the genes in *S. uvarum.* Growth curves, which were detected during the culture of yeast cells at 30 °C in YEPD medium, showed that the mutant strains exhibited a similar growth rate and final cell density as the parental strain. Weight losses were detected every 12 h during the fermentation process, and production of ethanol, residual sugar, and organic acid in wine were detected after AF. Table 4 and Figure 9 and Figure 10 suggest that the mutant strains with heterologous expressions of the genes had similar fermentation rate and no obvious changes in the liquor yield and residual sugar compared with the parental strain WY1.

In current study, we constructed a biological pathway of deacidification into *S. uvarum*, which make the *S. uvarum* carry out the AF and MLF simultaneously, as different genes related to the deacidification were heterologously expressed in *S. uvarum.* Analysis of L–malic acid production and other flavor substances (higher alcohols and esters) produced by the mutant strains with different gene expression and coexpression suggested different roles for the relevant enzymes during deacidification of AF in wine. In addition, of all the recombinant strains, the engineered *S. uvarum* that expressed gene *MAE1* from *S. pombe* and *mleS* from *L. lactis* exhibited the best effect, not only on deacidification of the wine, but also on the improvement in flavor quality. Our work settled several important questions caused by LAB in deacidification of MLF, and shortened the fermentation period.

## Figures and Tables

**Table 1:** Strains and plasmids used in the current study.

Strains or Plasmids	Relevant Characteristic	Reference or Source
Strain		
*E. coli* DH5α	supE44ΔlacU169(ϕ80lacZΔM15) hsdR17 recAl endAl gyrA96 thi-1 relA	This lab
WY1	Wild-type industrial *Saccharomyces uvarum*	This lab
*Lactococcus lactis*	Wild-type industrial *Lactococcus lactis*	This lab
*Lactobacillus plantarum*	Wild-type industrial *Lactobacillus plantarum*	This lab
*Oenococcus oeni*	Wild-type industrial *Oenococcus oeni*	This lab
*Schizosaccharomyces pombe*	Wild-type industrial *Schizosaccharomyces pombe*	This lab
WY0	*PGK1p-PGK1t-loxP-KanMX-loxP*	This study
WYE	*PGK1p-MAE1-PGK1t-loxP-KanMX-loxP*	This study
WYS_N_	*PGK1p-mleS_N_* (*Lactococcus lactis*)*-PGK1t-loxP-KanMX-loxP*	This study
WYS_Z_	*PGK1p-mleS_Z_* (*Lactobacillus plantarum*)*-PGK1t-loxP-KanMX-loxP*	This study
WYA_J_	*PGK1p-mleA_J_*(*Oenococcus oeni*)*-PGK1t-loxP-KanMX-loxP*	This study
WYm2	*PGK1p-MAE2*(*Schizosaccharomyces pombe*)*-PGK1t-loxP-KanMX-loxP*	This study
WYP_N_	*PGK1p-mleP_N_* (*Lactococcus lactis*)*-PGK1t-loxP-KanMX-loxP*	This study
WYP_Z_	*PGK1p-mleP_Z_*(*Lactobacillus plantarum*)*-PGK1t-loxP-KanMX-loxP*	This study
WYP_J_	*PGK1p-mleP_J_*(*Oenococcus oeni*)*-PGK1t-loxP-KanMX-loxP*	This study
WYm1	*PGK1p-MAE1*(*Schizosaccharomyces pombe*)*-PGK1t-loxP-KanMX-loxP*	This study
WYPE	*PGK1p-mleP_N_*(*Lactococcus lactis*)*-PGK1t-PGK1p-MAE1-PGK1t-loxP* *-KanMX-loxP*	This study
WYPS_N_	*PGK1p-mleP_N_*(*Lactococcus lactis*)*-PGK1t-PGK1p-mleS_N_*(*Lactococcus lactis*)*-PGK1t-loxP-* *KanMX-loxP*	This study
WYPS_Z_	*PGK1p-mleP_N_*(*Lactococcus lactis*)*-PGK1t-PGK1p-mleS_Z_*(*Lactobacillus plantarum*)*-PGK1t-loxP-* *KanMX-loxP*	This study
WYPA_J_	*PGK1p-mleP_N_*(*Lactococcus lactis*)*-PGK1t-PGK1p-mleA_J_*(*Oenococcus oeni*)*-PGK1t-loxP-* *KanMX-loxP*	This study
WYPm2	*PGK1p-mleP_N_*(*Lactococcus lactis*)*-PGK1t-PGK1p-MAE2*(*Schizosaccharomyces pombe*)*-PGK1t-loxP-* *KanMX-loxP*	This study
WYm1E	*PGK1p-MAE1*(*Schizosaccharomyces pombe*)*-PGK1t-PGK1p-MAE1-PGK1t-loxP* *-KanMX-loxP*	This study
WYm1S_N_	*PGK1p-MAE1*(*Schizosaccharomyces pombe*)*-PGK1t-PGK1p-mleS_N_*(*Lactococcus lactis*)*-PGK1t-loxP-* *KanMX-loxP*	This study
WYm1S_Z_	*PGK1p-MAE1*(*Schizosaccharomyces pombe*)*-PGK1t-PGK1p-mleS_Z_*(*Lactobacillus plantarum*)*-PGK1t-loxP-* *KanMX-loxP*	This study
WYm1A_J_	*PGK1p-MAE1*(*Schizosaccharomyces pombe*)*-PGK1t-PGK1p-mleA_J_*(*Oenococcus oeni*)*-PGK1t-loxP-* *KanMX-loxP*	This study
WYm1m2	*PGK1p-MAE1*(*Schizosaccharomyces pombe*)*-PGK1t-PGK1p-MAE2*(*Schizosaccharomyces pombe*)*-PGK1t-loxP-* *KanMX-loxP*	This study
Plasmids		
Yep352 pUG6	Ap^r^, cloning vector *E. coli*/*S. cerevisiae* shuttle vector, containing *Amp*^+^ and *loxP-KanMX-loxP* cassette	This lab This study
pPGK1	*E. coli*/*S. cerevisiae* shuttle vector, containing *Amp*^+^and *PGK1p* and *PGK1t*	This study
pSH-Zeocin	Zeor, Cre expression vector	This study

**Table 2:** Primers used in the present study.

Primers	Sequence (5′→3′)
PGK-U	CGCGGATCCTCTAACTGATCTATCCAAAACTG
PGK-D	ACGCGTCGACTAACGAACGCAGAATTTTCGAG
MAE-U	GAATTCCAGATCTCCTCGAGATGCTTAGAACCAGACTATCCG
MAE-D	TCTATCGCAGATCCCTCGAGCTACAATTGGTTGGTGTGCAC
MAE2-U	GAATTCCAGATCTCCTCGAGGCACGTGGACCGTCTTACC
MAE2-D	TCTATCGCAGATCCCTCGAGAGTTGATGAATAACAATAGGAGAAA
mleS_N_-U	GAATTCCAGATCTCCTCGAGATGCGTGCACATGAAATTT
mleS_N_-D	TCTATCGCAGATCCCTCGAGTTAGTACTCTGGATACCATTTAAGA
K(ApaI)-U	CCGCTAACAATACCTGGGCCCCAGCTGAAGCTTCGTACGC
K(ApaI)-D	GCACACGGTGTGGTGGGCCCGCATAGGCCACTAGTGGATCTG
MAE1-U	GAATTCCAGATCTCCTCGAGTTCATTTTCTCTCTTGGCCAC
MAE1-D	TCTATCGCAGATCCCTCGAGCTTTTGTCATGAAATCCCTCTTA
mleP_N_-U	GAATTCCAGATCTCCTCGAGATGAAAAAACTTAAAGAAACGA
mleP_N_-D	TCTATCGCAGATCCCTCGAGTTAATAAAAGAATCGTATAAGAATT
PGK(SmaI)-U	GTACCCGGGTCTAACTGATCTATCCAAAACTGA
PGK(SmaI)-D	GATCCCCGGGTAACGAACGCAGAATTTTC
mleS_Z_-U	GAATTCCAGATCTCCTCGAGATGACAAAAACTGCAAGTGAAAT
mleS_Z_-D	TCTATCGCAGATCCCTCGAGCTATTTGCTGATGGCCCG
mleA_J_-U	GAATTCCAGATCTCCTCGAGATGACAGATCCAGTAAGTATT
mleA_J_-D	TCTATCGCAGATCCCTCGAGATTAGTATTTCGGATCCCACT
YmleP_N_-U	ATGAAAAAACTTAAAGAAACGA
YmleP_N_-D	TTAATAAAAGAATCGTATAAGAATT
YmleS_Z_-U	ATGACAAAAACTGCAAGTGAAAT
YmleS_Z_-D	CTATTTGCTGATGGCCCG
YmleA_J_-U	ATGACAGATCCAGTAAGTATT
YmleA_J_-D	ATTAGTATTTCGGATCCCACT
K-U	CAGCTGAAGCTTCGTACGC
K-D	GCATAGGCCACTAGTGGATCTG
ApaI-U	CCTGCTTCAAACCGCTAACAATA
ApaI-D	CGAATGCACACGGTGTGGT
SmaI-U	TTCGAGCTCGGTACCCG
SmaI-D	AGTTAGAGGATCCCCGGG

**Table 3:** Contents of the higher alcohols and esters produced by the mutants and the parental strain in the study ^a^.

Strains	n-Propanol (mg/L)	Isobutyl Alcohol (mg/L)	Isoamyl Alcohol (mg/L)	Phenethyl Alcohol (mg/L)	Ethyl Acetate (mg/L)	Ethyl Lactate (mg/L)
WY1	51.83 ± 2.94	35.354 ± 1.54	201.53 ± 2.67	13.521 ± 0.98	23.301 ± 1.04	≈0
WY0	52.342 ± 3.212	35.421 ± 1.89	204.321 ± 2.85	13.395 ± 1.13	23.721 ± 1.75	≈0
WYE	51.921 ± 1.97	35.015 ± 2.16	205.127 ± 3.57	13.832 ± 0.97	23.55 ± 2.53	≈0
WYm2	52.044 ± 1.72	34.199 ± 1.05	212.105 ± 7.61 *	14.145 ± 1.32	28.521 ± 1.93 *	≈0
WYSN	50.697 ± 1.56	29.955 ± 3.52 *	176.282 ± 4.05 *	14.290 ± 1.53	21.716 ± 1.00 *	11.115 ± 0.59 *
WYAJ	51.017 ± 1.05	31.832 ± 1.35 *	189.205 ± 2.13 *	14.315 ± 1.05	21.573 ± 0.45 *	5.065 ± 0.84 *
WYSZ	50.538 ± 1.35	32.375 ± 2.30 *	191.372 ± 1.85 *	13.580 ± 1.38	21.063 ± 0.75 *	6.130 ± 0.05 *
WYP_N_	53.757 ± 2.45	36.335 ± 0.15 *	217.858 ± 5.93 *	14.033 ± 0.75	27.473 ± 1.89 *	≈0
WYm1	55.646 ± 0.89	45.244 ± 1.92 *	223.662 ± 3.19 *	14.257 ± 0.62	25.914 ± 2.33 *	≈0
WYPE	52.216 ± 3.67	40.785 ± 0.98 *	219.903 ± 4.97 *	13.889 ± 1.05	26.530 ± 1.83 *	≈0
WYPSN	52.385 ± 2.41	34.639 ± 1.53	210.775 ± 6.05 *	14.496 ± 1.42	29.741 ± 2.04 *	13.942 ± 1.42 *
WYPSZ	52.353 ± 2.86	35.745 ± 2.05	205.62 ± 3.55 *	13.753 ± 1.95	24.342 ± 1.05 *	7.431 ± 1.55 *
WYPAJ	51.964 ± 2.67	35.069 ± 1.80	204.175 ± 2.42	13.766 ± 1.65	20.156 ± 0.05	5.905 ± 1.80 *
WYPm2	54.791 ± 1.98	34.881 ± 2.94	211.031 ± 2.45 *	14.468 ± 1.08	29.896 ± 1.52 *	≈0
WYm1E	55.521 ± 4.05 *	45.004 ± 5.08 *	220.291 ± 3.12 *	14.823 ± 0.67	26.393 ± 2.74 *	≈0
WYm1SN	51.068 ± 1.54	28.184 ± 1.06 *	171.756 ± 1.09 *	14.630 ± 0.32	15.850 ± 1.08 *	13.992 ± 1.08 *
WYm1SZ	53.532 ± 2.05	34.977 ± 2.04	202.437 ± 4.96	12.966 ± 0.85	27.906 ± 2.48 *	9.327 ± 0.76 *
WYm1AJ	52.462 ± 1.77	35.728 ± 3.62	182.482 ± 4.96 *	12.964 ± 1.05	14.874 ± 2.16 *	5.594 ± 0.63 *
WYm1m2	53.705 ± 3.06	32.144 ± 2.95 *	210.968 ± 3.25 *	13.989 ± 1.93	32.705 ± 1.33 *	≈0

^a^ Data are the average of three independent experiments ± the standard deviation. Significant difference of the mutants and the parental strain was confirmed by Student’s *t*-test (* *p* < 0.05, n = 3).

**Table 4:** Fermentation performances of the mutant strains and the parental strain in the wine fermentation ^a^.

Strains	Weight Losses of CO_2_ (g)	Ethanol (%, *v*/*v*)	Residual Sugar (g/L)
WY1	14.95 ± 0.15	11.02 ± 0.12	2.65 ± 0.10
WY0	14.83 ± 0.25	11.03 ± 0.15	2.78 ± 0.21
WYE	14.90 ± 0.14	11.04 ± 0.15	2.66 ± 0.10
WYm2	14.79 ± 0.12	10.95 ± 0.20	2.70 ± 0.08
WYSN	14.82 ± 0.20	10.80 ± 0.14	2.60 ± 0.05
WYAJ	14.92 ± 0.22	11.16 ± 0.22	2.61 ± 0.12
WYSZ	14.93 ± 0.20	12.10 ± 0.25	2.78 ± 0.27
WYP_N_	14.72 ± 0.12	11.00 ± 0.15	2.67 ± 0.20
WYm1	14.79 ± 0.12	10.95 ± 0.18	2.62 ± 0.14
WYPE	14.75 ± 0.14	11.05 ± 0.21	2.7 ± 0.15
WYPSN	14.82 ± 0.20	10.95 ± 0.10	2.65 ± 0.15
WYPSZ	14.85 ± 0.15	11.05 ± 0.05	2.70 ± 0.02
WYPAJ	14.80 ± 0.22	11.00 ± 0.13	2.72 ± 0.13
WYPm2	14.79 ± 0.12	11.10 ± 0.15	2.66 ± 0.12
WYm1E	14.75 ± 0.14	11.00 ± 0.14	2.65 ± 0.15
WYm1SN	14.86 ± 0.24	11.05 ± 0.10	2.64 ± 0.14
WYm1SZ	14.83 ± 0.12	11.00 ± 0.06	2.70 ± 0.10
WYm1AJ	14.62 ± 0.15	10.83 ± 0.12 *	2.97 ± 0.05 *
WYm1m2	14.82 ± 0.20	11.02 ± 0.15	2.62 ± 0.06

^a^ Data are the average of three independent experiments ± the standard deviation. Significant difference of the mutants and the parental strain was confirmed by Student’s *t*-test (* *p* < 0.05, n = 3).

## Data Availability

The original contributions presented in the study are included in the article, further inquiries can be directed to the corresponding authors.
